# Accounting for culinary practices to improve FFQ-based vitamin C estimates in epidemiological studies: a cross-sectional analysis

**DOI:** 10.1007/s00394-026-04026-3

**Published:** 2026-06-29

**Authors:** Lucía Belzunce, Leticia Goni, Maria Soledad Hershey, M. Teresa Barrio-López, Pablo Ramos, Luis Tercedor, Jose Luis Ibáñez Criado, Alicia Ibañez Criado, Rosa Macías-Ruíz, Víctor de la O, Eduardo Castellanos, Ignacio García-Bolao, Jesús Almendral, Miguel Ruiz-Canela

**Affiliations:** 1 Department of Preventive Medicine and Public Health, University of Navarra—Instituto de Nutrición y Salud (INS), Pamplona, Spain; 2 Instituto de Investigación Sanitaria de Navarra (IdiSNA), Pamplona, Spain; 3 Fisiopatología de la Obesidad y Nutrición (CIBERObn), Instituto de Salud Carlos III, Madrid, Spain; 4 Human Flourishing Program, Institute for Quantitative Social Science, Harvard University, Cambridge, USA; 5 Electrophysiology Laboratory and Arrhythmia Unit, HM CIEC MADRID (Centro Integral de Enfermedades Cardiovasculares), Hospital Universitario HM Montepríncipe, HM Hospitales, Instituto de Investigación Sanitaria HM Hospitales, Madrid, Spain; 6 Arrhythmia Unit, Department of Cardiology and Cardiac Surgery, Clínica Universidad de Navarra, Pamplona, Spain; 7 Department of Cardiology, Virgen de las Nieves University Hospital, Granada, Spain; 8 Biosanitary Research Institute of Granada (ibs.GRANADA), Granada, Spain; 9 Arrhythmia Unit. Department of Cardiology, Dr. Balmis General University Hospital, Alicante Institute for Health and Biomedical Research (ISABIAL), Alicante, Spain; 10 Faculty of Health Sciences, International University of La Rioja (UNIR), Logroño, Spain; 11 Nutritional Control of the Epigenome Group, Precision Nutrition and Obesity Program, IMDEA Food, Campus of Excellence International, Universidad Autónoma de Madrid + Spanish National Research Council, Madrid, Spain

**Keywords:** Cooking methods, Ascorbic acid, Questionnaires

## Abstract

**Purpose:**

Conventional vitamin C intake estimations via food frequency questionnaires (FFQ) typically ignore cooking techniques, which can significantly alter nutrient content. This study evaluates the effect of incorporating a home cooking frequency questionnaire (HCFQ) alongside a FFQ to calculate vitamin C intake and its impact on adherence to dietary recommendations.

**Methods:**

We conducted a cross-sectional analysis in the PREDIMAR study, a randomized trial that aims to evaluate the effect of a Mediterranean diet supplemented with extra virgin olive oil on atrial fibrillation recurrence after ablation. Vitamin C intake was estimated in two ways: derived from (1) raw food using a FFQ and the Spanish food composition tables, and (2) from raw and cooked food using a FFQ+HCFQ. Adherence to estimated average requirements and a recommended intake of 200 mg/day were used to assess nutritional adequacy. Paired *t*-Test compared vitamin C mean intakes derived from both methods, and paired McNemar tests assessed differences in adequacy rates.

**Results:**

Among 447 patients (25.1% female; mean age = 59.7 years (10.1)), mean vitamin C intake (SD) was significantly higher when derived solely from the FFQ (173.7 mg/day (71.2)) vs. FFQ+HCFQ (160.1 mg/day (68.8); *p* < 0.001). The largest decreases of vitamin C, when cooking techniques were considered, were observed in legumes (− 88.0%), tubers (− 62.7%), and vegetables (− 12.5%). The main source of between-person variability in vitamin C intake from vegetables and tubers was raw vegetables and boiled potatoes, respectively. Adequacy of vitamin C intake significantly dropped using FFQ+HCFQ (21.5%) vs. FFQ alone (30.4%, *p* < 0.001).

**Conclusions:**

Ignoring cooking methods may lead to overestimation by approximately 8% of both vitamin C intake and adequacy prevalence in epidemiologic research. Incorporating a cooking-frequency questionnaire yields more conservative and potentially accurate estimates, improving nutritional epidemiology precision.

**Supplementary Information:**

The online version contains supplementary material available at 10.1007/s00394-026-04026-3.

## Introduction

Epidemiological studies frequently rely on food frequency questionnaires (FFQs) to estimate long-term dietary intake [1]. Nutrient intake is then estimated by applying food composition tables, which usually provide raw nutrient values [2]. However, cooking methods significantly impact nutrient content [3]. In addition to temperature, other factors such as the cooking medium (air or liquid), pH level, and cooking time influence the extent of nutrient retention and loss [4]. Water-based cooking methods (e.g., boiling) can lead to the release of water-soluble nutrients into the cooking liquid, whereas high-temperature cooking may result in nutrient degradation and the formation of toxic compounds such as polycyclic aromatic hydrocarbons (PAHs) and nitrosamines [5, 6].

One nutrient particularly susceptible to cooking loss is vitamin C, a heat and water-sensitive micronutrient abundant in fruits and vegetables [7]. Epidemiological evidence has shown that adequate vitamin C intake is associated with reduced risk of ischemic stroke, coronary heart disease in women, and esophageal cancer [8–10]. These effects are primarily attributed to vitamin C’s antioxidant capacity, which helps neutralize reactive oxygen species (ROS) [11]. While the estimated average requirements (EARs) for vitamin C are 75 mg/day and 60 mg/day for men and women, respectively [12]; higher intakes, around 200 mg/day, have been suggested to maximize health benefits [13]. In 2018, the global prevalence of inadequate vitamin C intake was estimated at 4.0 billion people, with South and East Asia and the Pacific being the regions with the highest prevalence, particularly among males [14]. Additionally, a review of factors influencing vitamin C levels also described a higher prevalence of deficiency among South Asians, possibly due to traditional cooking practices [15].

Because FFQs fail to account for cooking modifications, they may systematically over- or underestimate actual nutrient intake. To address this measurement limitation, a Home Cooking Frequency Questionnaire (HCFQ) was recently developed and validated, allowing the integration of consumption frequency with the types and frequencies of cooking methods used at home [16]. In our previous 2023 study, combining FFQ and HCFQ yielded estimates of advanced glycation end products (AGEs) that showed a stronger correlation with plasma AGE levels than FFQ alone [17].

In the present study, we aimed to assess how incorporating cooking techniques using both the FFQ and HCFQ affects vitamin C intake estimates and the percentage of individuals meeting adequacy thresholds based on EARs and recommended optimal intake levels. In addition, we aimed to evaluate the contribution of each cooking technique to the main dietary sources of vitamin C and to between-person variability.

## Materials and methods

### Study population

We conducted a cross-sectional analysis within the PREDIMAR (Prevención con Dieta Mediterránea de Arritmias Recurrentes) study, a randomized, controlled, multicenter, single-blind trial designed to evaluate the effect of a Mediterranean Diet (MedDiet) supplemented with extra virgin olive oil (EVOO) on reducing atrial fibrillation (AF) recurrence in patients undergoing ablation. The study protocol has been previously published [18]. Briefly, participants were recruited between 2017 and 2020 at 4 Spanish hospitals: Hospital Montepríncipe (Madrid), Clínica Universidad de Navarra (Pamplona), Hospital Virgen de las Nieves (Granada), and Hospital General Universitario de Alicante (Alicante). Inclusion criteria were patients with symptomatic paroxysmal or persistent AF undergoing ablation. Participants were randomly assigned to either the usual care (control group) or the dietary intervention (MedDiet supplemented with EVOO) group, which was remotely delivered and coordinated by the Department of Preventive Medicine and Public Health at the University of Navarra [19]. The nutritional intervention lasted for 2 years, followed by 2 years of extended follow-up without intervention. Clinical and lifestyle data were collected at baseline and during the follow-up at months 3, 6, 12, 18, 24, 36, and 48.

The PREDIMAR trial is registered in ClinicalTrials.gov NCT03053843. This study was conducted according to the guidelines laid down in the Declaration of Helsinki, and all procedures involving research study participants were approved by the Research Ethics Committees of each recruitment center. All participants provided written informed consent after they received the information sheet and additional verbal explanation of the study characteristics.

### Assessment of dietary and culinary habits

**Fig. 1:**
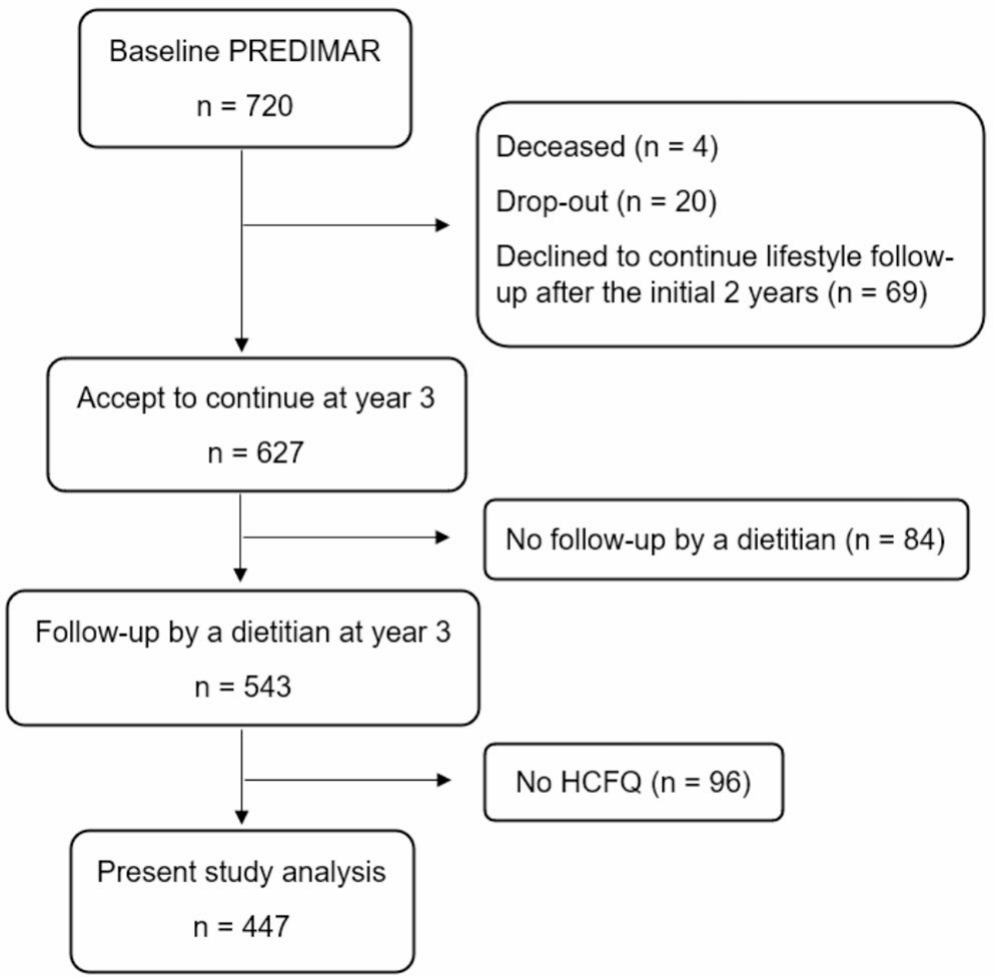
Flowchart of the participants in the PREDIMAR study. HCFQ, Home cooking frequency questionnaire

Data used in the present study were collected via telephone by a trained dietitian-nutritionist during the 3-year follow-up visit. A total of 720 participants started the PREDIMAR study. After completion of the 2-year intervention, participants were invited to extend follow-up for an additional 2 years. Of the 627 participants who agreed to continue, only 447 completed both, the FFQ and the HCFQ (Fig. 1).

A previously validated semi-quantitative FFQ in the Spanish population, based on Willett methodology, was used to assess dietary habits [20]. This FFQ comprises a total of 147 items with 9 possible consumption frequencies for a defined portion size (never or < 1 time/month, 1–3 times/month, 1 time/week, 2–4 times/week, 5–6 times/week, 1 time/day, 2–3 times/day, 4–6 times/day, and > 6 times/day). The Spanish food composition tables were used to assess energy and nutrient intake [21, 22]. For items encompassing multiple foods, an average of their contributions was calculated.

Culinary habits and cooking methods were analyzed at the 3-year follow-up using a recently validated HCFQ in a Spanish population [16]. The HCFQ comprises 171 items categorized into five domains (cooking habits, dietary habits, cooking techniques, cooking ingredients and cooking utensils). For this study, the cooking techniques domain was utilized to estimate cooking-related nutrient modifications. This domain captures frequency of 8 techniques response options (never or almost never, 1–3 times/month, 1–2 times/week, 3–4 times/week, 5–6 times/week, 1 time/day, 2 times/day, and 3 or more times/day) for 9 food groups (eggs, white meat, red meat, fish and seafood, vegetables, tubers, fruits, legumes, and cereals).

The average time required to complete each questionnaire separately was approximately 20 min. However, because both questionnaires were administered together and by a trained dietitian-nutritionist, the total completion time was reduced to approximately 30 min.

### Measurement of other variables

Lifestyle and sociodemographic variables were collected at baseline: age, sex, educational level, working status, weight and height, and smoking habit. Adherence to the MedDiet and physical activity were collected. Adherence to the MedDiet was measured annually using the 14-item Mediterranean Diet Adherence Screener (MEDAS), a previously validated questionnaire [23], and physical activity was assessed using a physical activity questionnaire validated in the Spanish population [24].

### Vitamin C intake from the FFQ and the FFQ+HCFQ

Vitamin C intake based on the FFQ was calculated using the raw values from the Centre d’Ensenyament Superior de Nutrició i Dietètica (CESNID) food composition tables, chosen because these Spanish tables include many foods also in their cooked form [25]. The estimation was based exclusively on dietary sources, without considering potential vitamin C supplementation. All foods quantified in the FFQ could be matched to their corresponding items in these tables; therefore, no alternative sources were required to obtain vitamin C raw values. To determine vitamin C intake according to FFQ+HCFQ, cooking-related retention factors were applied to raw CESNID values. These retention factors were derived from an integrated database built using multiple sources, selected according to the following hierarchical decision algorithm. For each food item and cooking technique, a single retention factor was assigned based on the highest-priority source available. First, Spanish food composition tables were used [25]. If a cooking technique for a food was not available in these tables, French tables were used as the second option [26], followed by American tables as a third option [27]. When no information was available in any of these sources, food composition tables from the EuroFIR database were used [28]. Subsequently, peer-reviewed literature on vitamin C retention by cooking method were consulted [6, 7, 29–33]. When data were obtained from review articles, the mean retention value across the studies included in the review was used. Finally, missing values were estimated by applying nutrient retention factors published by the USDA [34] or, when direct data were unavailable, by using retention factors from similar culinary methods or from another food grouped within the same FFQ item. This yielded a customized retention factor table, including the corresponding reference (Table 1). These retention factors were applied to the CESNID vitamin C raw values to estimate cooked vitamin C content for each food-cooking method estimation [25].

**Table 1:**
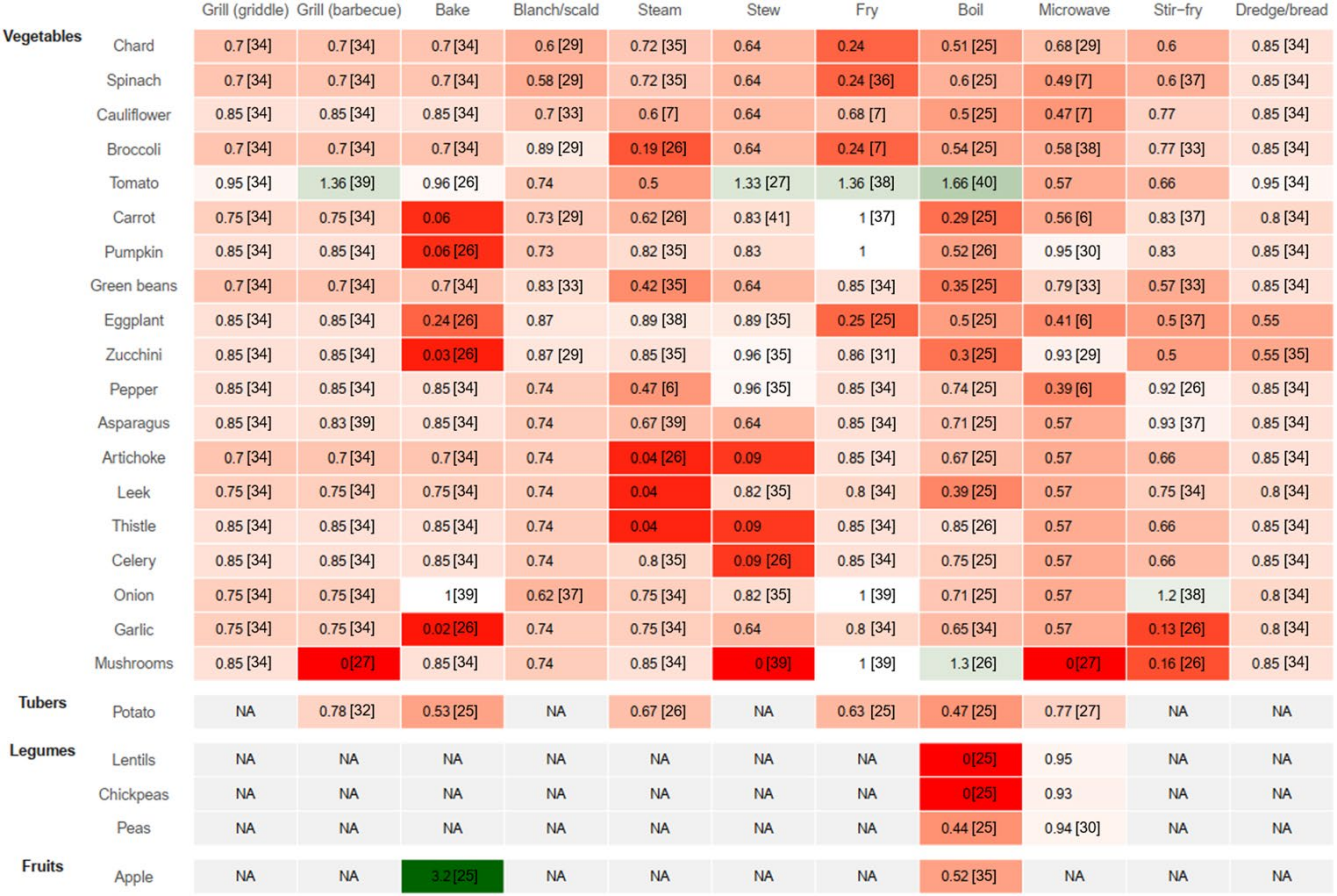
Theoretical retention factors of vitamin C based on the culinary techniques used

A secondary analysis was carried out applying only the nutrient retention factors from the USDA Tables [34]. This approach minimized differences in vitamin C intake estimations that could arise from variability among different bibliographic sources. For this method, the most similar cooking technique to the one referenced in the HCFQ was selected and applied.

Vitamin C intake derived from the FFQ was estimated for each participant multiplying the food intake (g/day) by the vitamin C content (mg/100 g of food). For the FFQ+HCFC method, vitamin C intake was calculated using the following formula:

Food intake (g/day) x Vitamin C content (mg/100 g of food) after cooking x Frequency of use of the culinary technique.

Vitamin C intake was summed across food groups, and total vitamin C intake was calculated for each culinary technique.

### Statistical analysis

Continuous variables are presented as median and interquartile range (IQR) or mean and standard deviation (SD), as specified in each table, whereas categorical variables are presented as frequencies and percentages. The two-sample Wilcoxon rank-sum test for continuous variables and Pearson’s χ^2^ test for categorical were used to compare differences between PREDIMAR participants included and excluded in the present analysis. Six participants presented energy intake values above Willett’s cut-off limits. However, no errors or unusual data collection patterns were identified, and all participants were therefore retained in the analysis.

Differences in vitamin C intake derived from the FFQ and the FFQ+HCFQ were analyzed using the paired Student’s t-Test in the main analyses and the Wilcoxon signed-rank test in the supplementary analyses. Additionally, the percentage of vitamin C intake difference was calculated, both overall and according to food groups.

The percentage contribution of vitamin C intake from vegetables and tubers, the highest contributors of vitamin C, was estimated based on each cooking technique and raw consumption, as these food groups are frequently consumed cooked and particularly affected by cooking processes. Since 99% of vitamin C intake from fruits comes from raw consumption, no cooking techniques were applied to this food group, except for boiled and baked apples, which were the only cooked fruit preparations considered. However, their contribution to total fruit intake, and particularly to vitamin C intake, was very low. All legume consumption was estimated as being cooked (boiled).

The percentage of vitamin C provided by each cooking method to the total intake was calculated. We quantified the contribution of each cooking technique to between-person variability in total vitamin C intake using a Shapley decomposition of the coefficient of determination (R^2^). We fitted linear regression models with total vitamin C intake from vegetables (and separately, tubers) as the dependent variable and technique-specific vitamin C intakes (mg/day) as predictors. We then computed Shapley values for R^2^ in Stata (shapley2, stat(r2)), defined as the average marginal increase in R^2^ attributable to each technique across all possible predictor orderings. Shapley contributions were reported as percentages of the total explained variance providing an order-invariant measure of relative importance.

Finally, to assess nutritional adequacy of vitamin C intake, we applied two reference thresholds: (1) the EARs of 75 mg/day men and 60 mg/day women, defined as intake levels covering the requirements of 50% of healthy individuals in a given age and sex-defined group [12], and (2) the optimal recommended intake of 200 mg/day intended to maximize vitamin C’s protective health effects [13]. We calculated the proportion of participants meeting each threshold under both FFQ-only and FFQ + HCFQ derived estimates. Differences in adequacy classifications between the two methods were analyzed using the paired McNemar test. In addition, as a secondary analysis, vitamin C intake adequacy was stratified by sex.

Statistical analyses were performed using Stata/MP version 14.0 (StataCorp, College Station, TX, USA) software package. All *p* values were two-tailed, and a *p* value < 0.05 was considered statistically significant.

## Results

Baseline sociodemographic, anthropometric and lifestyle-related characteristics for the total population of the PREDIMAR study, as well as a comparison between participants included and not included in this cross-sectional analysis, are presented in Table 2. Among the 447 participants included, 25.1% were women and the median baseline age (IQR) was 60.7 (53.4; 67.2) years. Statistically significant differences were observed: Included participants more frequently belonged to the intervention group, were married, had paroxysmal AF, and had university-level education, and they also reported higher physical activity levels than those not included.

**Table 2:** Baseline characteristics of the PREDIMAR study participants

Characteristics	Total population (*n* = 720)^a^	Included population (*n* = 447)^a^	Non-included population (*n* = 273)^a^	*p* value^b^
Age (years)	60.9 (53.3; 67.6)	60.7 (53.4; 67.2)	61.2 (53.2; 68.5)	0.544
BMI (kg/m^2^)	27.2 (24.9; 30.1)	27.3 (24.9; 30.2)	27.2 (25.0; 30.1)	0.962
Physical activity (MET-h/week)	33.6 (17.3; 43.4)	33.6 (18.2; 45.7)	32.6 (17.1; 41.7)	0.032
Sex, women	171 (23.8)	112 (25.1)	59 (21.6)	0.292
Intervention group				
Control	355 (49.3)	204 (45.6)	151 (55.3)	0.012
Intervention	365 (50.7)	243 (54.4)	122 (44.7)	
AF type				
Paroxysmal	431 (59.9)	282 (63.1)	149 (54.6)	0.024
Persistent	289 (40.1)	165 (36.9)	124 (45.4)	
Married				
Yes	553 (76.8)	360 (80.5)	193 (70.7)	0.002
No	167 (23.2)	87 (19.5)	80 (29.3)	
University				
Yes	320 (44.4)	215 (48.1)	105 (38.5)	0.012
No	400 (55.6)	232 (51.9)	168 (61.5)	
Working status				
Working	390 (54.2)	233 (52.1)	157 (57.5)	0.369
Retired	272 (37.8)	176 (39.4)	96 (35.2)	
Others	58 (8.1)	38 (8.5)	20 (7.3)	
Smoking				
Never	260 (36.1)	169 (37.8)	91 (33.3)	0.147
Current	46 (6.4)	23 (5.2)	23 (8.4)	
Former	414 (57.5)	255 (57.1)	159 (58.2)	

AF, Atrial Fibrillation; BMI, Body mass index; MET, Metabolic equivalent of task

^a^ Continuous variables: median (IQR), categorical variables: frequency (percentage)

^b^ Differences in baseline characteristics between included and non-included population

Table 3 shows dietary and cooking habits at the 3-year follow-up among the study population. The mean (SD) energy intake was 2223 (567) kcal/day, with macronutrient distribution of 39.0% (7.0) carbohydrates, 41.7% (6.3) fats and 16.3% (2.6) proteins. Mean MedDiet adherence score, according to the MEDAS questionnaire (0–14 points) was 8.9 (2.1) points. A total of 17.5% of the participants planned weekly menus, and 82.8% handled the weekly grocery shopping. Among the 309 (69.1%) participants who reported cooking at home, 32.4% practiced batch cooking, and 65.1% cooked at least once per day. Boiling was the most frequently used culinary technique (8.5 times per week), while grilling (barbecue) was the least employed (0.1 times per week).

**Table 3:** Dietary and culinary habits of the participants included in the present study at the third-year follow-up visit

	Included population (*n* = 447) Median (IQR)	Included population (*n* = 447) Mean ± SD
Dietary habits		
Energy intake (kcal/day)	2157.9 (1837.4; 2519.3)	2223 ± 566.6
Carbohydrate intake (% of energy)	38.7 (34.6; 42.8)	39.0 ± 7.0
Fiber intake (g/day)	23.0 (18.7; 28.8)	24.4 ± 8.3
Fat intake (% of energy)	41.7 (37.9; 45.7)	41.7 ± 6.3
Protein intake (% of energy)	16.3 (14.6; 18.0)	16.3 ± 2.6
MedDiet adherence (0–14 points)^a^	9 (7; 10)	8.9 ± 2.1
Culinary techniques frequency use		
Grill (griddle/pan)	4.4 (2.9; 6.0)	4.4 ± 2.7
Grill (barbecue)	0 (0; 0)	0.1 ± 0.4
Roast in the oven	2.0 (0.5; 3.5)	2.4 ± 2.1
Steam	0 (0; 0.5)	0.8 ± 1.6
Stew	1.4 (0.5; 2.0)	1.5 ± 1.5
Fry	2.0 (0.5; 3.0)	2.2 ± 1.8
Boil	8.0 (6.5; 10.4)	8.5 ± 3.1
Microwave	0 (0; 0)	0.2 ± 0.6
Dredge and bread	0 (0; 0.5)	0.3 ± 0.7
Stir-fry	0 (0; 1.5)	0.8 ± 1.2
Raw	3.5 (3.5; 7.0)	4.4 ± 2.9
Culinary habits^b^		
Do you plan weekly menus at home?		
Yes, I am in charge of weekly meal planning	78 (17.5)	
No, but we do a weekly meal plan at home	47 (10.5)	
We do not do a weekly meal plan	322 (72.0)	
Are you in charge or do you participate in weekly grocery shopping?, yes	371 (82.8)	
Do you cook at home?, yes	309 (69.1)	
Do you do batch cooking? (*n* = 309), yes	100 (32.4)	
How many days do you spend cooking each week? (*n* = 309)		
< 7 days/week	108 (35.0)	
7 days/week	201 (65.1)	
How many hours do you spend cooking each week? (*n* = 309)		
< 7 h	183 (59.2)	
> 7 h	126 (40.8)	

MedDiet, Mediterranean Diet; SD, Standard deviation; IQR, Interquartile range

^a^ Mediterranean diet adherence screener (MEDAS)^24^

^b^ Frequency (percentage)

Table 4 presents total and food group-specific vitamin C intake estimates derived from the FFQ alone and the FFQ+HCFQ. The vitamin C intake based on the FFQ alone was significantly higher (173.7 (71.2) mg/day) than the calculated using the FFQ+HCFQ (157.3 (68.0) mg/day) (*p* < 0.001). Statistically significant differences were also observed across all food groups (all *p* values < 0.05). The largest reductions were seen in legumes (88.0%), tubers (62.7%), and vegetables (12.5%), whereas, as expected, fruits remained virtually unchanged (+ 0.1%) when using the FFQ+HCFQ. These findings are consistent with those observed when non-parametric tests were used (Supplementary Material Table 1). In the secondary analysis using only the USDA tables retention factors (Supplementary Material Table 2), vitamin C intake remained significantly lower (160.1 (68.8) mg/day) than the estimated intake from raw foods (*p* < 0.001), resulting in an 8.5% mean difference between derived methods. When stratified by food groups, the mean vitamin C intake after cooking was also significantly lower, except for fruits, which maintained the same vitamin C content as that derived from the FFQ. In this secondary analysis, tubers remained the most affected food group (46.5% difference), followed by legumes (34.3% difference), and then vegetables (12.0% difference). The non-parametric analyses yielded comparable results (Supplementary Material Table 3).

**Table 4:** Vitamin C intake (mg/day), total and by food groups, according to the FFQ and the FFQ+HCFQ (Parametric analysis)

Vitamin C intake from	FFQ Mean ± SD	FFQ + HCFQ Mean ± SD	*p* value	% difference Mean ± SD
Total food	173.7 ± 71.2	157.3 ± 68.0	< 0.001	− 10.1 ± 5.6
Vegetables	60.2 ± 29.2	52.4 ± 24.8	< 0.001	− 12.5 ± 7.7
Legumes	0.9 ± 0.9	0.2 ± 0.4	< 0.001	− 88.0 ± 16.8
Potato and other tubers	12.4 ± 7.7	4.5 ± 2.9	< 0.001	− 62.7 ± 11.6
Fruits	77.9 ± 48.9	78.0 ± 49.0	0.034	+ 0.1 ± 1.2
Dairy products	3.2 ± 2.5	3.2 ± 2.5 ^a^	NA	NA
Liver and other offal	0.2 ± 0.5	0.2 ± 0.5 ^a^	NA	NA
Cereals	1.6 ± 5.0	1.6 ± 5.0 ^b^	NA	NA
Juices	16.3 ± 28.1	16.3 ± 28.1 ^b^	NA	NA
Miscellaneous	0.7 ± 0.8	0.7 ± 0.8 ^b^	NA	NA

FFQ, Food Frequency Questionnaire; HCFQ, Home Cooking Frequency Questionnaire; NA, Not applicable; SD, Standard deviation

^a^ The HCFQ not include these products or lack of information on the available data

^b^ Foods mostly consumed raw or processed

^c^ Miscellaneous includes pizzas, croquettes, commercial tomato sauce, mustard, and jam

Table 5 presents vitamin C intake from vegetables and tubers by cooking technique (FFQ+HCFQ estimates), presented in decreasing order of use. This analysis was not performed for vitamin C from fruits or legumes because approximately 99% of vitamin C intake was derived from raw fruits and boiled legumes, respectively. For vegetables, 51.5% of the vitamin C was attributed to raw consumption, and 15.4% to boiled vegetables, the most common cooking technique. For tubers (e.g. potatoes), boiled potatoes, also the most frequently used cooking technique, accounted for 35.9% of the vitamin C intake, and baked preparations contributed 24.2%. In both cases, the lowest contribution came from grilled vegetables and potatoes.

**Table 5:** Vitamin C intake (mg/day) from vegetables and tubers, according to type of culinary technique, ordered from most to least frequently used, and between-person variability

Vegetables	Vitamin C (mg/day) Median (IQR)	Vitamin C (mg/day) Mean ± SD	Shapley* contribution to *R*^2^, %	Tubers	Vitamin C (mg/day) Median (IQR)	Vitamin C (mg/day) Mean ± SD	Shapley contribution to *R*^2^, %
Raw	24.7 (15.6; 34.0)	26.2 ± 16.2	51.5%	Boil	1.3 (0.6; 3.3)	2.1 ± 2.0	35.9%
Boil	11.1 (5.5; 18.4)	12.6 ± 10.4	15.4%	Roast in the oven	0.3 (0; 1.4)	1.0 ± 1.5	24.2%
Grill (griddle/pan)	0 (0; 6.1)	4.2 ± 7.8	10.8%	Fry	0.3 (0; 1.1)	0.9 ± 1.5	13.1%
Stir-fry	0 (0; 6.5)	4.0 ± 7.3	6.9%	Steam	0 (0; 0)	0.3 ± 1.3	12.7%
Roast in the oven	0 (0; 0)	1.7 ± 5.6	6.5%	Microwave	0 (0; 0)	0.3 ± 1.2	12.4%
Steam	0 (0; 0)	1.5 ± 4.4	4.0%	Grill (barbecue)	0 (0; 0)	0.04 ± 0.5	1.7%
Stew	0 (0; 0)	1.2 ± 4.5	3.1%				
Blanch and scald	0 (0; 0)	0.09 ± 1.4	0.9%				
Fry	0 (0; 0)	0.5 ± 3.0	0.6%				
Other techniques**	0 (0; 0)	0.2 ± 1.3	0.3%				

SD, Standard deviation; IQR, Interquartile range

* Shapley contributions are expressed as the percentage of the model R^2^ attributable to each cooking technique (order-invariant), computed from regression models using technique-specific vitamin C intakes (mg/day) as predictors

**Microwave, Dredge and bread, and grill (barbecue)

Finally, Table 6 summarizes nutritional adequacy of dietary intake vitamin C according to FFQ versus FFQ + HCFQ methods, assessed using both EAR and the optimal 200 mg/day benchmark. The prevalence of nutritional inadequacy under EARs standards (75 mg/day for men and 60 mg/day for women), was significantly lower when using the FFQ (3.4%) compared to the FFQ+HCFQ (6.9%). When analyses were stratified by sex (Supplementary material Table 4), statistically significant differences between the FFQ and the FFQ+HCFQ method were observed only in men, not in women. Using the optimal intake of 200 mg/day, 30.4% of participants met the recommendations using the FFQ alone, versus only 21.5% when using the FFQ+HCFQ method. In sex-stratified analyses, statistically significant differences between the two methods were observed in both men and women (Supplementary material Table 5).

**Table 6:** Differences in the adequacy of food-based vitamin C intake derived from the FFQ or the FFQ+HCFQ

	EAR (75 mg/day ♂, 60 mg/day ♀) Frequency (%)		Recommended intake (200 mg/day) Frequency (%)	
	FFQ	FFQ + HCFQ	FFQ	FFQ + HCFQ
Meet	432 (96.6%)	416 (93.1%)	136 (30.4%)	96 (21.5%)
Do not meet	15 (3.4%)	31 (6.9%)	311 (69.6%)	351 (78.5%)
*p* value	< 0.001		< 0.001	

FFQ, Food Frequency Questionnaire; HCFQ, Home Cooking Frequency Questionnaire; EAR, Estimated average requirements

## Discussion

This study found statistically significant differences in vitamin C intake derived from the FFQ alone versus the FFQ+HCFQ combination. Notably, more than 50% of vitamin C from vegetables was sourced from raw consumption, meanwhile for tubers, nearly 36% originated from boiled potatoes. Raw vegetables and boiled potatoes emerged as the primary contributors to between-person variability in vitamin C intake. Additionally, the prevalence of vitamin C nutritional inadequacy was significantly higher when vitamin C intake was derived using the FFQ+HCFQ compared to the FFQ alone.

Previous studies have established the importance of considering cooking techniques when studying the relationship between diet and health, given their significant impact on nutritional composition [42]. However, food composition tables and previous studies often lack consistent or complete nutritional information on cooked foods. In fact, we found large variations between the raw values provided by different bibliographic sources for the same foods [6, 7, 25–27, 29–33, 35–41]. This is consistent with previous findings reported by Willet in Nutritional Epidemiology, where he described how nutrient content varies depending on factors such as harvesting conditions, degree of maturity, size, storage, and climate [43]. Thus, more research is needed along with the development of updated food composition tables that provide accurate nutrient composition data for individual foods after applying different culinary techniques.

Our theoretical retention factors suggested that baking, steaming and stewing were particularly detrimental to vitamin C retention (Table 1). Steaming was identified as one of the most aggressive methods for vitamin C according to the literature sources used in this study [26]. This is consistent with the findings of another study, which reported a 32% loss using this technique [31]. However, it contradicts other studies that describe steaming as one of the most effective techniques for preserving nutrients due to its minimal use of water [33]. For example, one study found that steaming was the best method for preserving vitamin C in broccoli [44]. These discrepancies may be due to differences in the steaming equipment and cooking times used across studies. The other most affected cooking techniques were baking and stewing, which aligns with recent evidence. Baking exposes food to high temperatures, leading to nutrient degradation [45]. Meanwhile stewing is similar to boiling, which has been shown to cause a significant loss of vitamin C [7]. Interestingly, an increase in vitamin C levels was observed in tomatoes following grilling, stewing, frying, and boiling. Some studies have reported increases in certain lycopene isomers in tomatoes when exposed to heat and combined with other ingredients such as olive oil, onions, and garlic to cook a sofrito [46]. While this phenomenon has not been studied specifically for vitamin C, our findings suggest that a similar process could occur, given that vitamin C is also an antioxidant [47]. Further research is needed to better understand the effects of cooking techniques on food composition and to establish more consistent methodologies for reliable conclusions.

Vitamin C losses were higher in legumes, a food group typically cooked extensively. While few studies have evaluated the effect of cooking techniques on vitamin C, other water-soluble and heat-sensitive vitamins, such as B1, B2 and B6, have been documented and vary depending on cooking time and temperature [48]. In our cohort, vegetables retained half of their vitamin C from raw consumption, possibly due to high MedDiet adherence, which often prescribes daily servings consumed raw [49]. In contrast, potatoes must be cooked to improve their digestibility, resulting in greater vitamin C losses than in vegetables. These losses may differ between potatoes varieties though, cooking them with peel may help reduce vitamin C degradation [50]. We found that boiling was the most frequently used cooking technique contributing to vitamin C intake, for both vegetables and tubers. Similar results were observed in a study with U.S. population, where boiling was the preferred method among those who cooked vegetables [51]. Another study conducted in a Spanish population found that the most common culinary techniques were grilling, stewing and baking [52]. These results are consistent with those found in our study, as grilling was the second most common technique used for vegetables, while baking was predominant for potatoes.

Retention factors were not applied to fruits; therefore, vitamin C content estimates were similar between the FFQ alone and the FFQ+HCFQ approach. The only cooked fruit consumption considered was boiled and baked apples, as these were the most frequently consumed cooked fruit preparations in the study sample. However, their contribution to total vitamin C intake remained very low, since 99% of fruit-derived vitamin C came from raw consumption. This is consistent with the latest Spanish food consumption data reported by the AESAN (Agencia Española de Seguridad Alimentaria y Nutrición), which show that fruit is predominantly consumed fresh [53].

By not accounting for culinary techniques, some individuals may be misclassified in epidemiological studies as meeting vitamin C requirements when they actually do not. Numerous epidemiological studies have found an inverse association between vitamin C intake and the incidence of chronic diseases, such as cancer [10] and cardiovascular disease [8]. However, other studies have either not found a significant association or have only observed it in the context of vitamin C supplementation, rather than dietary intake [9, 54], despite strong biological mechanisms supporting vitamin C’s health benefits [55]. It has been hypothesized that a suboptimal adjustment for potential confounders, such as fiber intake, could explain the inverse association found in some studies between vitamin C and cardiovascular disease incidence. However, in the Seguimiento Universidad de Navarra (SUN) cohort, an inverse association between vitamin C intake and cardiovascular mortality was observed even after adjustment for total fiber intake [56]. Based on our findings, this inconsistency could be explained by a possible classification bias. Additionally, in studies where a significant inverse association was found, the magnitude of the effect may be underestimated, due to misclassification of individuals in the high-intake group that could skew the results toward a null effect [8, 10]. Prospective studies and randomized clinical trials should consider integrating cooking techniques into dietary assessment to better capture nutrient content and reduce misclassification in exposure measurement.

A primary strength of this study is the use of previously validated questionnaires [16, 20], which were administered by trained dietitian-nutritionists. Another strength was the use of multiple sources of scientific literature to develop the most comprehensive vitamin C retention factors database. One limitation was the lack of comprehensive nutritional data on cooked foods, particularly for less common culinary techniques applied to specific vegetables. However, this was addressed by drawing from a wide range of scientific literature. A second limitation was the high variability in vitamin C values reported across different food composition tables and studies for the same foods. To minimize potential information bias arising from inconsistencies in food composition sources, retention factors were calculated and applied to the raw values of vitamin C intake used in the FFQ, thereby aligning estimates more closely with CESNID raw values and improving internal consistency with national food composition standards. Also, the secondary analysis which only considered the USDA retention factors showed similar results to those found in the main analysis. Another limitation related to the HCFQ is that it collects data on the frequency of cooking techniques used for each food group rather than individual foods, assuming that the frequency of each culinary technique is the same for all foods within a group. Additionally, because total vitamin C intake is closely related to the sum of technique-specific intakes (mg/day), the overall model R^2^ is expected to be very high; thus, Shapley estimates should be interpreted as a descriptive partition of explained variance (relative importance). Vitamin C intake was assessed considering only dietary sources and not supplements, which may have underestimated vitamin C adequacy prevalence. However, the proportion of participants who used supplements was very low (less than 5%), and the objective of the study was to evaluate the effect of culinary techniques on nutrients provided by food. The cross-sectional design of the study represents a limitation, as it precludes establishing temporality and does not allow causal inferences to be drawn. Therefore, the observed associations between culinary techniques applied to foods and vitamin C nutritional adequacy should be interpreted with caution. Longitudinal studies are needed to better assess potential cause-and-effect relationship. The time required to complete the questionnaire could also be considered a limitation. However, because many of the questions have dichotomous answer (yes/no) and the questions regarding the frequency of cooking techniques are repeated across food groups, the time required is not excessively long. Moreover, as the questionnaire was administered by trained dietitian-nutritionists and together with the FFQ, the total time required to complete both questionnaires was reduced. Therefore, the HCFQ can be considered a valuable tool to complement the information provided by the FFQ. In addition, because the HCFQ encompasses a broad range of culinary techniques commonly used in numerous countries, it could be adapted for use in other countries following appropriate translation and validation processes. However, differences in culinary habits across countries may influence the findings related to vitamin C intake, highlighting the need for further validation studies in diverse populations. Finally, this study lacked blood biomarkers, such as plasma vitamin C, which could provide an objective measure of vitamin C intake. However, several studies have shown that the correlation between nutrient intake and certain metabolites is not always strong [57].

## Conclusions

This study suggests that vitamin C intake may be overestimated when derived from a FFQ alone, without accounting for nutrient losses from cooking techniques. Incorporating a HCFQ alongside a FFQ data captures cooking-related degradation and yields more accurate vitamin C intake and prevalence of inadequacy. Adoption of such combined methods in nutritional epidemiology may reduce misclassification bias in epidemiological studies and strengthen associations between vitamin C intake and health outcomes. Further research, including prospective cohorts and trials that account for cooking practices, is warranted to inform dietary and cooking recommendations as well as public health nutrition strategies.

## Supplementary Information

Below is the link to the electronic supplementary material.

Supplementary Material 1

## Data Availability

The data underlying this article will be shared on reasonable request to the corresponding author.
