# Supplementary material for: Accounting for culinary practices to improve FFQ-based vitamin C estimates in epidemiological studies: a cross-sectional analysis
Source: Eur J Nutr. 2026 Jun 29;65(5):185. doi: 10.1007/s00394-026-04026-3 (PMC13315505; doi:10.1007/s00394-026-04026-3)
Supplement: Supplementary file 1 — Supplementary Material 1 [file 394_2026_4026_MOESM1_ESM.docx]

**ARTICLE TITLE**

Accounting for Culinary Practices to Improve FFQ-Based Vitamin C Estimates in Epidemiological Studies: a cross-sectional analysis

**JOURNAL NAME**

European Journal of Nutrition

**AUTHORS’ NAMES**

Lucía Belzunce^1^, Leticia Goni^1,2^, Maria Soledad Hershey^3^, M. Teresa Barrio-López^4^, Pablo Ramos^5^, Luis Tercedor^6,7^, Jose Luis Ibáñez Criado^8^, Alicia Ibañez Criado^8^, Rosa Macías-Ruíz ^6^, Víctor de la O^9,10^, Eduardo Castellanos^4^, Ignacio García-Bolao^5^, Jesús Almendral^4^, Miguel Ruiz-Canela^1,2^

**CORRESPONDING AUTHOR**

Miguel Ruíz Canela, PhD., MPH, Pharm.D.

University of Navarra, Department of Preventive Medicine and Public Health C/ Irunlarrea, 31008 Pamplona, Spain

Affiliation:

Department of Preventive Medicine and Public Health, University of Navarra—IdiSNA (Instituto de Investigacion Sanitaria de Navarra), Pamplona, Spain

Fisiopatología de la Obesidad y Nutrición (CIBERObn), Instituto de Salud Carlos III, Madrid, Spain

Email: [mcanela@unav.es](mailto:mcanela@unav.es)

| **Supplementary Table 1.** Vitamin C intake (mg/day), total and by food groups, according to the FFQ and the FFQ+HCFQ (Non-parametric analysis) | | | | |  |
| --- | --- | --- | --- | --- | --- |
| Vitamin C intake from | FFQ  Median (IQR) | FFQ + HCFQ  Median (IQR) | p value | % difference  Median (IQR) | |
| Total food | 162.6 (124.3; 213.0) | 144.8 (112.6; 192.8) | <0.001 | -9.2 (-12.4; -6.3) | |
| Vegetables | 54.4 (41.6; 69.4) | 47.9 (36.4; 60.4) | <0.001 | -11.6 (-16.6; -7.5) | |
| Legumes | 0.5 (0.3; 1.5) | 0 (0; 0.6) | <0.001 | -100 (-100; -68.8) | |
| Potato and other tubers | 9.5 (6.6; 18.2) | 2.9 (2.2; 6.8) | <0.001 | -67.4 (-71.6; -52.6) | |
| Fruits | 72.8 (43.0; 98.5) | 72.8 (43.0; 98.5) | 0.007 | 0 (0; 0) | |
| Dairy products | 3.0 (1.4; 3.9) | 3.0 (1.4; 3.9) ^a^ | NA | NA | |
| Liver and other offal | 0 (0; 0) | 0 (0; 0) ^a^ | NA | NA | |
| Cereals | 0 (0; 0) | 0 (0; 0) ^b^ | NA | NA | |
| Juices | 0.1 (0; 27.4) | 0.1 (0; 27.4) ^b^ | NA | NA | |
| Miscellaneous | 0.5 (0.1; 0.9) | 0.5 (0.1; 0.9) ^b^ | NA | NA | |
| FFQ, Food Frequency Questionnaire; HCFQ, Home Cooking Frequency Questionnaire; NA, Not applicable; IQR, Interquartile range  ^a^ The HCFQ not include these products or lack of information on the available data  ^b^ Foods mostly consumed raw or processed  ^c^ Miscellaneous includes pizzas, croquettes, commercial tomato sauce, mustard, and jam. | | | | |  |

| **Supplementary Table 2.** Vitamin C intake (mg/day), total and by food groups, according to FFQ and FFQ+HCFQ (secondary analysis^a^) (Parametric analysis) | | | | |
| --- | --- | --- | --- | --- |
|  | FFQ  Mean ± SD | FFQ + HCFQ  Mean ± SD | p value | % difference  Mean ± SD |
| Total food | 173.7 ± 71.2 | 160.1 ± 68.8 | <0.001 | -8.5 ± 4.4 |
| Vegetables | 60.2 ± 29.2 | 52.9 ± 25.6 | <0.001 | -12.0 ± 5.3 |
| Legumes | 0.9 ± 0.9 | 0.6 ± 0.6 | <0.001 | -34.3 ± 16.8 |
| Potato and other tubers | 12.4 ± 7.7 | 6.5 ± 4.1 | <0.001 | -46.5 ± 15.2 |
| Fruits | 77.9 ± 48.9 | 77.9 ± 48.9 | 0.001 | -0.03 ± 0.2 |
| Dairy products | 3.2 ± 2.5 | 3.2 ± 2.5 ^b^ | NA | NA |
| Liver and other offal | 0.2 ± 0.5 | 0.2 ± 0.5 ^b^ | NA | NA |
| Cereals | 1.6 ± 5.0 | 1.6 ± 5.0 ^c^ | NA | NA |
| Juices | 16.3 ± 28.1 | 16.3 ± 28.1 ^c^ | NA | NA |
| Miscellaneous ^d^ | 0.7 ± 0.8 | 0.7 ± 0.8 ^c^ | NA | NA |
| FFQ, Food Frequency Questionnaire; HCFQ, Home Cooking Frequency Questionnaire; NA, Not applicable; SD, Standard deviation  ^a^  Using only the USDA tables retention factors  ^b^ The HCFQ not include these products or lack of information on the available data  ^c^ Foods mostly consumed raw or processed  ^d^ Miscellaneous includes pizzas, croquettes, commercial tomato sauce, mustard, and jam. | | | | |

| **Supplementary Table 3.** Vitamin C intake (mg/day), total and by food groups, according to FFQ and FFQ+HCFQ (secondary analysis^a^) (Non-parametric analysis) | | | | |
| --- | --- | --- | --- | --- |
|  | FFQ  Median (IQR) | FFQ + HCFQ  Median (IQR) | p value | % difference  Median (IQR) |
| Total food | 162.6 (124.3; 213.0) | 148.0 (113.0; 198.0) | <0.001 | -7.6 (-10.5; -5.4) |
| Vegetables | 54.4 (41.6; 69.4) | 47.7 (36.5; 60.5) | <0.001 | -11.4 (-14.7; -8.8) |
| Legumes | 0.5 (0.3; 1.5) | 0.4 (0.2; 1.1) | <0.001 | -30 (-30; -30) |
| Potato and other tubers | 9.5 (6.6; 18.2) | 4.1 (3.5; 10.2) | <0.001 | -52.7 (-59.7; -32.8) |
| Fruits | 72.8 (43.0; 98.5) | 72.8 (43.0; 98.5) | <0.001 | 0 (0; 0) |
| Dairy products | 3.0 (1.4; 3.9) | 3.0 (1.4; 3.9) ^b^ | NA | NA |
| Liver and other offal | 0 (0; 0) | 0 (0; 0) ^b^ | NA | NA |
| Cereals | 0 (0; 0) | 0 (0; 0) ^c^ | NA | NA |
| Juices | 0.1 (0; 27.4) | 0.1 (0; 27.4) ^c^ | NA | NA |
| Miscellaneous ^d^ | 0.5 (0.1; 0.9) | 0.5 (0.1; 0.9) ^c^ | NA | NA |
| FFQ, Food Frequency Questionnaire; HCFQ, Home Cooking Frequency Questionnaire; NA, Not applicable; IQR, Interquartile range  ^a^  Using only the USDA tables retention factors  ^b^ The HCFQ not include these products or lack of information on the available data  ^c^ Foods mostly consumed raw or processed  ^d^ Miscellaneous includes pizzas, croquettes, commercial tomato sauce, mustard, and jam. | | | | |

| **Supplementary Table 4.** Differences in the adequacy of vitamin C intake, according to the EAR, derived from the FFQ or the FFQ+HCFQ and stratified by sex | | | | |
| --- | --- | --- | --- | --- |
| EAR  Frequency (%) | | | | |
|  | Males  (75 mg/day) | | Women  (60 mg/day) | |
|  | FFQ | FFQ+HCFQ | FFQ | FFQ+HCFQ |
| Meet | 322 (96.12%) | 308 (91.94%) | 110 (98.21%) | 108 (96.43%) |
| Do not meet | 13 (3.88%) | 27 (8.06%) | 2 (1.79%) | 4 (3.57%) |
| p value | 0.0001 | | 0.5000 | |

| **Supplementary Table 5.** Differences in the adequacy of vitamin C intake, according to the recommended intake, derived from the FFQ or the FFQ+HCFQ and stratified by sex | | | | |
| --- | --- | --- | --- | --- |
| Recommended intake (200 mg/day)  Frequency (%) | | | | |
|  | Males | | Women | |
|  | FFQ | FFQ+HCFQ | FFQ | FFQ+HCFQ |
| Meet | 87 (25.97%) | 64 (19.10%) | 49 (43.75%) | 32 (28.57%) |
| Do not meet | 248 (74.03%) | 271 (80.90%) | 63 (56.25%) | 80 (71.43%) |
| p value | 0.0001 | | <0.001 | |

| **Supplementary Table 6**. Variation in vitamin C content of raw and boiled cauliflower, broccoli, and peppers across bibliographic sources | | | |
| --- | --- | --- | --- |
| Food | Cooking technique | Reference | Vitamin C content |
| Cauliflower | Raw | Spanish tables | 50 |
|  |  | French tables | 4.14 (92.9 max) |
|  |  | American tables | 48.2 |
|  | Boil | Spanish tables | 25 |
|  |  | American tables | 44.3 |
| Broccoli | Raw | Spanish tables | 110 |
|  |  | French tables | 106 |
|  |  | American tables | 91.3 |
|  | Boil | Spanish tables | 60 |
|  |  | French tables | 23.9 (64.9 max) |
|  |  | American tables | 64.9 |
| Peppers | Raw | Spanish tables | 134.5 |
|  |  | French tables | 121 |
|  |  | American tables | 130.8 |
|  | Boil | Spanish tables | 99 |
|  |  | French tables | 77.5 |
|  |  | American tables | 123 |

**Sample calculation of vitamin C intake by the FFQ+HCFQ method**

1st. Vitamin C intake from grilled (pan) vegetables was calculated as:

(frequency of consumption of each vegetable * vitamin C content under this cooking technique * portion size/100g * correction factor * (frequency of consumption of grilled vegetables/frequency of consumption of vegetables)

Stata code:

generate vitC_grilledvegetables= (chard * 26.25 * 2 * 0.7+ cauliflower * 59.75 * 2 * 0.7+ lettuce * 12 * 1 * 0.7+ tomato * 18.1 * 1.5 * 0.7+ carrot * 7.75 * 1 * 0.7+ greenbeans * 14 * 2 * 0.7+ eggplant * 10.2 * 2 * 0.7+ pepper * 114.3 * 1.5 * 0.7 +asparagus * 11.9 * 2 * 0.7 + gazpacho * 4.8 * 2 * 0.7+ greensalad * 6.98 * 2 * 0.7+ onion * 5.3 * 0.5 * 0.7+ garlic * 16.5 * 0.03 * 0.7+ mushrooms * 3.4 * 2 * 0.7)* grilledvegetables /totalcookedvegetables

2nd. The same calculation was repeated for each culinary technique assessed for vegetables in the HCFQ.

3rd. Total vitamin C intake from vegetables derived from the FFQ and HCFQ was calculated as the sum of vitamin C intake from: raw vegetables, boiled vegetables, pan-grilled vegetables, stir-fried vegetables, roasted vegetables, stewed vegetables, steamed vegetables, fried vegetables, blanched/scalded vegetables, dredged/breaded vegetables, barbecued vegetables, and microwaved vegetables.

4th. The same calculation was applied to legumes, potatoes and other tubers, and fruits.

5th. Total vitamin C intake from FFQ and HCFQ was calculated as the sum of vitamin C intake from vegetables, potatoes and other tubers, fruits, and vitamin C intake from dairy products, liver and other offal, cereals, juices and miscellaneous food derived from FFQ.
